# Diversity of Symbiodiniaceae in 15 Coral Species From the Southern South China Sea: Potential Relationship With Coral Thermal Adaptability

**DOI:** 10.3389/fmicb.2019.02343

**Published:** 2019-10-18

**Authors:** Zhenjun Qin, Kefu Yu, Biao Chen, Yinghui Wang, Jiayuan Liang, Wenwen Luo, Lijia Xu, Xueyong Huang

**Affiliations:** ^1^Coral Reef Research Center of China, Guangxi University, Nanning, China; ^2^Guangxi Laboratory on the Study of Coral Reefs in the South China Sea, Nanning, China; ^3^School of Marine Sciences, Guangxi University, Nanning, China; ^4^College of Forestry, Guangxi University, Nanning, China

**Keywords:** coral, zooxanthellae density, Symbiodiniaceae subclade, interspecific variation, thermal stress adaptability

## Abstract

It is well-known that the adaptability of coral-Symbiodiniaceae symbiosis to thermal stress varies among coral species, but the cause and/or mechanism behind it are not well-understood. In this study, we aimed to explore this issue based on zooxanthellae density (ZD) and Symbiodiniaceae genus/subclade. Hemocytometry and next-generation sequencing of the internal transcribed spacer region 2 (ITS2) marker gene were used to observe ZDs and Symbiodiniaceae genera/subclades associated with 15 typical coral species in the southern South China Sea (SCS). Average ZDs of all corals were in low levels, ranging from 0.84 to 1.22 × 10^6^ cells cm^−2^, with a total of five Symbiodiniaceae genera, *Symbiodinium, Cladocopium, Durusdinium, Fugacium*, and *Gerakladium*, as well as 24 dominant subclades, were detected and varied among these coral species. *Pocillopora verrucosa* was dominated by *Durusdinium* (subclade D1/D1a), and other colonial corals were dominated by *Cladocopium*, but the subclades were varied among these species. *Porites lutea* and *Montipora efflorescens* were dominated by C15, and *Echinopora lamellosa, Hydnophora exesa*, and *Coscinaraea exesa* were dominated by C40. *Acropora corymbosa, Merulina ampliata*, and five species of Faviidae were mainly associated with *Cladocopium* types of C3u and Cspc. In contrast to other colonial corals, the dominant subclade of solitary *Fungia fungites* was C27, with high host specificity. Our study indicates that coral thermal stress adaptability is mainly affected by dominant Symbiodiniaceae type instead of ZD in the southern SCS. Some heat-sensitive corals, such as *P. verrucosa* corals, have acquired a high abundance of heat-tolerant *Durusdinium* to adapt to thermal stress. This could be the main reason for these corals becoming the dominant corals in this reef region. Background subclades analyses showed significant differences among coral species in subclade quantity and diversity. These suggest that numbers of coral species may have adapted to high environmental temperature by adopting various symbionts and/or associating with heat-tolerant Symbiodiniaceae.

## Introduction

Coral reef ecosystems, with high biodiversity and productivity, are facing severe threats from anthropogenic disturbance and global warming (Brown, 1997; Bellwood et al., 2004; Yu, 2012). Reef corals rely on their symbiotic relationship with Symbiodiniaceae (i.e., zooxanthellae), which provide ~95% of their energy and enable them to develop in oligotrophic tropical seas (Falkowski et al., 1984). Abnormally elevated sea surface temperatures (SSTs) disrupt this symbiosis, resulting in zooxanthellae discharge and coral death (Baker et al., 2004, 2008). Since the 1990s, coral reefs around the world have frequently experienced global-scale bleaching events, mainly including the 1998, 2010, and 2015/2016 global thermal bleaching events (e.g., Hoegh-Guldberg, 1999; Loya et al., 2001; Hughes et al., 2017, 2018). The 1998 thermal bleaching event caused great damage to global coral reefs. More than 16% of global coral reefs were lost−87% of corals in the inshore reefs of the Great Barrier Reef were bleached, ~95% of corals died after bleaching in Bahrain and the Maldives, and ~85% of corals were bleached and lost in Japan (Wilkinson, 1998; Berkelmans and Oliver, 1999; Loya et al., 2001). Record high temperatures during 2015/2016 triggered the third recorded global bleaching event since the first large-scale bleaching in the 1980s, causing severe harm to the survival and health of global coral reefs (Hughes et al., 2017, 2018).

Experimental and *in situ* studies have found that corals' tolerance to thermal stress varies widely among species (Marshall and Baird, 2000; Abrego et al., 2008; Wicks et al., 2010; Wooldridge, 2014). In natural conditions, branching *Acropora* corals with low zooxanthellae densities (ZDs) are vulnerable to thermal bleaching, while massive *Favia* and *Porites* corals with high ZDs have higher tolerance to thermal stress (Li et al., 2008, 2011). Branching *Acropora* and *Pocillopora* corals are the dominant colonial species in the tropical coral reefs of the Indo-Pacific and are considered to be highly vulnerable to thermal bleaching (Loya et al., 2001; Wooldridge, 2014). In 1998, these species suffered severely from a bleaching event and a large number died (Berkelmans and Oliver, 1999; Marshall and Baird, 2000; Loya et al., 2001). Since that time, subsequent global bleaching events have occurred (Moore et al., 2012; Hughes et al., 2017; Nohaïc et al., 2017). Surprisingly, a number of coral genera/species (e.g., *Porites* and *Montastraea* corals) survive after repeated thermal stress, indicating that these corals may have begun to develop increased thermal adaptability (Pratchett et al., 2013; Silverstein et al., 2015; Boulotte et al., 2016; Guest et al., 2016). Previous studies have shown that zooxanthellae density is an important indicator of coral susceptibility to thermal stress (Marshall and Baird, 2000; Li et al., 2008; Wooldridge, 2014; Xu et al., 2017). However, due to limited reports on its diversity and abundance, it is difficult to assess whether density is a factor affecting its potential for adaptation to climate change. Further research into intraspecific variations in coral tolerance and adaptability to thermal stress can help to shed light on this question.

Recent studies suggest that dominant Symbiodiniaceae clades in coral-algal symbionts are closely associated to coral bleaching tolerance (Ortiz et al., 2012; Hume et al., 2015; Silverstein et al., 2015). Symbiodiniaceae have been revised and divided into a series of genera, comprising *Symbiodinium, Breviolum, Cladocopium, Durusdinium, Effrenium, Fugacium, Gerakladium*, formerly described as Clades A, B, C, D, E, F, and G, respectively (Pochon et al., 2004; Pochon and Gates, 2010; LaJeunesse et al., 2018). In addition, these genera can be identified in different subclades based on next-generation sequencing (NGS). Numerous subclades have been identified using high-resolution molecular markers in rDNA internal transcribed spacer regions ITS1 and/or ITS2 (e.g., LaJeunesse and Trench, 2000; LaJeunesse, 2001; Arif et al., 2014), the hypervariable regions of domain V of the chloroplast large subunit (cp23S) (e.g., Santos et al., 2002; Pochon et al., 2006; LaJeunesse et al., 2018), and the chloroplast psbA non-coding region (psbA^ncr^) (e.g., LaJeunesse and Thornhill, 2011; Reimer et al., 2017). Among these markers, the ITS2 region remains the most used marker for analyzing the community and diversity of Symbiodiniaceae (e.g., Pochon et al., 2014; Cunning et al., 2017; Smith et al., 2017b). The phenotypes of coral hosts of Symbiodiniaceae types confer different physiological features and thermal stress tolerance (Brading et al., 2011; Hume et al., 2015, 2016). For example, the symbionts dominant with *Durusdinium* generally have higher thermal tolerance than those that are primarily associated with other clades (Berkelmans and Oppen, 2006; Mostafavi et al., 2007; Jones et al., 2008; Sampayo et al., 2008). Therefore, corals hosting unique or multiple symbiotic Symbiodiniaceae genera/subclades can be expected to have varying abilities to deal with environmental stress (Sampayo et al., 2008; Silverstein et al., 2015).

Investigating the diversity and function of Symbiodiniaceae offers insight into their response to thermal stress (LaJeunesse et al., 2003; Baker et al., 2004; Jones et al., 2008). Recent applications of novel techniques such as quantitative PCR and NGS have yielded increasing information on previously unknown low-abundance Symbiodiniaceae genera and subclades (Bay et al., 2016; Ziegler et al., 2017, 2018). In coral symbiotic microbial ecosystems, rare biospheres represent low-abundance and highly diverse clades accounting for <1%, which are generally referred to as background types (Thomas et al., 2014; Ziegler et al., 2017). Background Symbiodiniaceae subclades are important for coral-symbionts' adaptation to thermal stress (Boulotte et al., 2016; Lee et al., 2016; Ziegler et al., 2018). Coral hosts under such stress might accommodate Symbiodiniaceae by either symbiosis “reorganization” or “transformation” (Fautin and Buddemeier, 2004; Apprill and Gates, 2010; Cunning et al., 2016). Some coral species, such as *Pocillopora damicornis* and *Stylophora pistillata*, have been shown to resist and/or recover from thermal stress by altering the relative abundance of Symbiodiniaceae types (Baker et al., 2004; Silverstein et al., 2015; Boulotte et al., 2016; Ziegler et al., 2018). However, the role of background Symbiodiniaceae in dealing with thermal stress is insufficiently understood and requires further exploration, particularly of their composition and diversity.

The Spratly Islands (i.e., Nansha Islands, 3°35′-11°55′N, 109°30′-117°50′E) are located in the southernmost area of the South China Sea (SCS), with typical tropical reefs near the equator (Yu, 2012). The area has high diversities of scleractinian corals with high genetic diversity and connectivity, which are associated with a variety of microorganisms (Zhao et al., 2013; Liang et al., 2017; Huang et al., 2018). Previous macro-ecological surveys in the central and southern SCS have found that massive *Porites* and *Montipora* dominate, with additional prevalence of heat-sensitive branching *Pocillopora* corals (Zhao et al., 2013, 2016). They are affected by abnormal high temperature at times, and numerous of corals bleached under thermal stress (Yu and Zhao, 2006; Li et al., 2011; Yu, 2012). In this study, two fundamental issues were addressed: (i) we currently do not know the density, diversity, and composition of Symbiodiniaceae in numerous coral species and the main influence environmental factors in the southern SCS, and (ii) under the frequent threat of thermal bleaching events in recent decades, why the branching *Pocillopora* corals, which are generally considered to be heat-sensitive, are still dominant in the southern SCS? We tested the hypotheses that: (i) there would be clear differences in ZDs and Symbiodiniaceae genera among coral species in the southern SCS that would be shaped by local environmental factors, and (ii) the branching *Pocillopora* would have high ZDs or be associated with heat-tolerant Symbiodiniaceae genera to enhance their resistance to thermal stress and to be dominant in this coral reef region (CRR). To explore these questions—the ZDs and Symbiodiniaceae genera/subclades of 15 typical scleractinian coral species—local water quality parameters were collected at Xinyi Reef of the Spratly Islands. This study identifies the ZDs and Symbiodiniaceae genera/subclades among fifteen coral species and the main affecting factors. The results indicate variation in the ZDs and Symbiodiniaceae genera/subclades, and the potential relationship with bleaching susceptibility to the local environment stresses among coral species. Our findings contribute to further understanding the variation in adaptation to thermal stress among coral species under global warming.

## Materials and Methods

### Study Site and Sampling of Corals

Our study site was located at Xinyi Reef (9°20′6″N, 115°55′49″E) of the Spratly Islands, southern SCS (Figure S1). This is a small tropical atoll (~6.8 km^2^), and corals are mainly distributed on reef flats and slopes. Mean annual SST is relatively high at 28.7°C, with a monthly range from 27.4°C in January to 31.7°C in June (Figure S2). SSTs were obtained from satellite-derived datasets of NASA, Ocean Color Radiometry, monthly averaged MODIS-Aqua 9 km, from January 1997 to December 2015 (https://giovanni.gsfc.nasa.gov). Seawater temperature, salinity and transparency were measured during sampling. Dissolved oxygen (DO) was assessed *in situ* using a DO200A portable meter (YSI Inc., Yellow Springs, OH, USA). Seawater samples were collected and immediately filtered (Whatman GF/F; GE Healthcare, Chicago, IL, USA). Dissolved inorganic nitrogen (DIN) and soluble reactive phosphorus (SRP) were measured using a continuous flow analyzer (SEAL QuAAtro; SEAL Analytical Shanghai, Shanghai, China). Forty-eight coral specimens from eight families and 15 genera, including *Pocillopora verrucosa, Acropora corymbosa, Montipora efflorescens, Pavona varians, Echinopora lamellosa, Merulina ampliata, Porites lutea, Favia palauensis, Favites abdita, Goniastrea aspera, Diploastrea heliopora, Platygyra daedalea, Hydnophora exesa, Coscinaraea exesa*, and *Fungia fungites*, were randomly collected in three to five replicates (~30 cm^2^ each sample) at 2–6 m water depth (Table S1). These species contained four morphologies, i.e., branching corals, massive corals, plating corals, and solitary corals. The distance between replicates was above 10 m, and *in situ* photography was carried out at the same time to verify species identification. Each specimen was divided into two portions, where one (~25 cm^2^) was used to determine ZD and the other (~5 cm^2^) was used for Symbiodiniaceae DNA extraction. All specimens were preserved at 0–4°C and immediately transported to the laboratory.

### Determination of Zooxanthellae Density

Coral tissue was removed using a Waterpik^TM^ (3–5 kgf cm^−2^) containing seawater (passed through a 0.45 μm filter) until only white coral skeleton remained (1–3 min). At this point, the entire tissue was considered to be completely removed. To reduce the error as far as possible, all the experiments with ZDs were conducted by the same experimenter. Initial slurry volume was measured in a graduated cylinder. The slurry was then homogenized and subsampled into four 3-mL aliquots, then centrifuged at 4000 rpm for 5 min. ZDs were calculated using replicate hemocytometer counts (*n* = 8). Surface area was determined based on the correlation between aluminum foil weight and surface area (Marsh, 1970; Fitt et al., 2000; Li et al., 2008).

### DNA Extraction, PCR Amplification and Illumine MiSeq Sequencing

Coral samples of ~50 mg, including tissue, mucus, and skeleton, were used to extract genomic DNA using DNeasy® Plant Mini Kit (Qiagen, Hilden, Germany) following the manufacturer's protocol provided with the kit. After quality and purity examinations, the extracted DNA samples were applied as PCR templates. The Symbiodiniaceae ITS2 region of rDNA was amplified by PCR using primers F: 5′-GAATTGCAGAACTCCGTG-3′ and R: 5′-GGGATCCATATGCTTAAGTTCAGCGGGT-3′, with a six-nucleotide barcode unique to each sample (LaJeunesse and Trench, 2000; LaJeunesse et al., 2003). PCR amplifications were conducted in a 50 μL reaction volume containing ~50 ng of DNA, 25 μL of 2X Taq Platinum PCR Master (Tiangen, China), 200 nM of each primer, and ddH_2_O to make up the final volume. Reactions were performed at 94°C for 5 min, followed by 35 cycles of 94°C for 30 s, 51°C for 30 s, and 72°C for 30 s, and a final extension at 72°C for 5 min using a ABI GeneAmp® 9700 thermocycler as described by Sun et al. (2014). Triplicate PCR products were pooled for each sample, and fragments with size in range of 301 to 340 bp were purified and quantified using the AxyPrep DNA gel extraction kit (Axygen Biosciences, Union City, CA, United States) and QuantiFluorTM-ST Fluorescence quantitative system (Promega, United States). Purified amplicons were pooled in equimolar amounts and paired-end sequenced (2 × 250) on an Illumina MiSeq platform according to standard protocols (Majorbio Bio-Pharm Technology Co. Ltd., Shanghai, China). Raw reads were deposited into the NCBI Sequence Read Archive (SRA) database (Accession Number: SRP 186860).

### Next-Generation Sequencing Data Processing and Data Analysis

Strict quality control and sequence filtration were applied to ensure analytical accuracy. Adaptors, short reads, and low-quality reads were removed by the sequencing company. A paired-end read merger (PEAR) tool was applied to obtain full-length ITS2 rDNA fragments with merging overlapping PE reads to generate ITS2 sequences (Zhang et al., 2014). ITS2 tags were de-multiplexed into all samples in the QIIME platform by identifying unique barcodes (Caporaso et al., 2010). A BLAST Symbiodiniaceae-specific database of ITS2 types was downloaded (Supplementary Materials ITS2 Database.fasta) (Franklin et al., 2012; Arif et al., 2014; Chen et al., 2019). Sequences were assigned to the ITS2 types that gave the highest identity in the BLAST hits (Altschul et al., 1990). To evaluate Symbiodiniaceae diversity and community composition, ITS2 sequence data alignment analysis and OTU analysis were used in this study (Arif et al., 2014; Tong et al., 2017; Ziegler et al., 2017). In addition, dominant/sub-dominant Symbiodiniaceae genera/subclades (≥1%) were analyzed both by ITS2 sequence data alignment analysis and OTU analysis, and the background subclades (<1%) were analyzed by ITS2 sequence data alignment analysis (Arif et al., 2014; Tong et al., 2017; Ziegler et al., 2017). The resulting counts of Symbiodiniaceae ITS2 types were merged for downstream statistical analysis.

Inter-species variations in ZDs were investigated using one-way ANOVA after the assumptions testing of homogeneity, normality, and independence. The Student-Newman-Keuls (SNK) test was used for *post hoc* multiple comparisons of significant ANOVA results. All ZDs data are presented as means ± standard deviations (SD). Shannon's diversity index (H'), which is based on the OTU framework, was calculated to assess the level of alpha-diversity across samples in the Vegan package by R (v. 3.1.2) software environment (Dixon, 2003; R Core Team, 2014). The difference of Symbiodiniaceae diversity and community composition among the 15 coral species was conducted by Wilcoxon rank-sum test. The similarity of Symbiodiniaceae assemblages was also characterized using non-metric multidimensional scaling (nMDS) using the Bray-Curtis distance metric after data transformation (Tong et al., 2017). Two phylogenetic trees were constructed, with one for dominant/sub-dominant Symbiodiniaceae subclades (>1%) and the other for background subclades (0.1 to 1%), based on the Kimura 2-parameter model with uniform rates among sites using Maximum Likelihood in MEGA 6 and Bayesian inference in MrBayes (Ronquist et al., 2012; Tong et al., 2017).

## Results

### Local Environment Condition

Several seawater quality parameters, including temperature, salinity, transparency, turbidity, pH, DO, DIN, and SRP, were measured in this study. Seawater temperature, salinity, transparency, turbidity, pH, DO, DIN, and SRP in this CRR were 30.4 ± 0.6°C, 33.1 ± 0.8, 24.3 ± 2.5 m, 0.2 ± 0.03 NTU, 8.24 ± 0.14, 6.85 ± 0.04, 1.38 ± 0.15 μmol L^−1^, and 0.06 ± 0.02 μmol L^−1^, respectively (Tables S2, S3).

### Zooxanthellae Density

**Figure 1:**
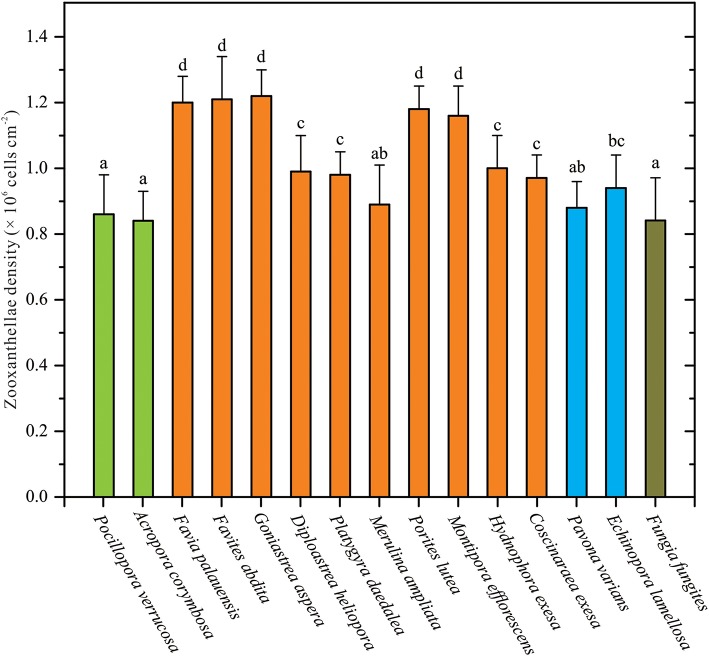
Corals' species-specific differences in zooxanthellae densities (ZDs). ZD data are given as mean ± SD. Letters above histograms denote statistical differences among these 15 coral species (Student-Newman-Keuls multiple range tests, *p* < 0.05). The colors of bars are meaning different coral morphologies, i.e., green bars meaning branching corals (*Pocillopora verrucosa* and *Acropora corymbosa*), orange-yellow bars meaning massive corals (e.g., *Favia palauensis, Porites lutea*), sky-blue bars meaning plating corals (*Pavona varians* and *Echinopora lamellosa*), and cyan bars meaning solitary corals (*Fungia fungites*).

The ZDs of 15 coral species were collected and measured in this study. Among these coral samples, average ZDs ranged from 0.84 to 1.22 × 10^6^ cells cm^−2^ (Table S4). There were significant variations in ZDs between coral species (one-way ANOVA, *p* < 0.05; Figure 1). ZD was highest in *G. aspera* at 1.22 ± 0.08 × 10^6^ cells cm^−2^, followed by *F. abdita, F. palauensis, P. lutea, M. efflorescens*, and *H. exesa*, ranging from 1.00 to 1.21 × 10^6^ cells cm^−2^. ZDs in *P. varians, D. heliopora, P. daedalea, M. ampliata, C. exesa*, and *E. lamellosa* ranged from 0.88 to 0.97 × 10^6^ cells cm^−2^, respectively, and the lowest densities were observed in *A. corymbosa* (0.84 ± 0.09 × 10^6^ cells cm^−2^), *P. verrucosa* (0.86 ± 0.12 × 10^6^ cells cm^−2^), and *F. fungites* (0.84 ± 0.13 × 10^6^ cells cm^−2^). Compared with the data from the northern SCS (Li et al., 2008; Xu et al., 2017), we found that ZDs in all these coral species were in low levels.

### Diversity and Composition of Coral-Associated Symbiodiniaceae Based on ITS2 Sequence Analysis

In total, 2,143,488 high-quality sequences were obtained from 48 samples (30,083 to 64,362 sequences per sample, Table S5). Based on ITS2 database alignments, a total of 188 Symbiodiniaceae ITS2 subclades were assigned, including *Symbiodinium, Cladocopium, Durusdinium, Fugacium, Gerakladium*, and clade I. Based on OTU analysis, Symbiodiniaceae ITS2 sequences were clustered into 24 OTUs at 97% similarity. These OTUs belonged to three Symbiodiniaceae genera (i.e., 18 OTUs in *Cladocopium*, 5 OTUs in *Durusdinium*, and 1 OTU in *Gerakladium*). At genus level, *P. verrucosa* had a high proportion of *Durusdinium* (average 90.6%), while *Cladocopium* was dominant in the other 14 species (average >85%, Table S6). *Gerakladium* was detected in some corals (e.g., *P. lutea, M. ampliata*, and *P. daedalea*), but was not dominant (<10%), followed by rare clades A, F, and I (<1%, Table S6).

**Figure 2:**
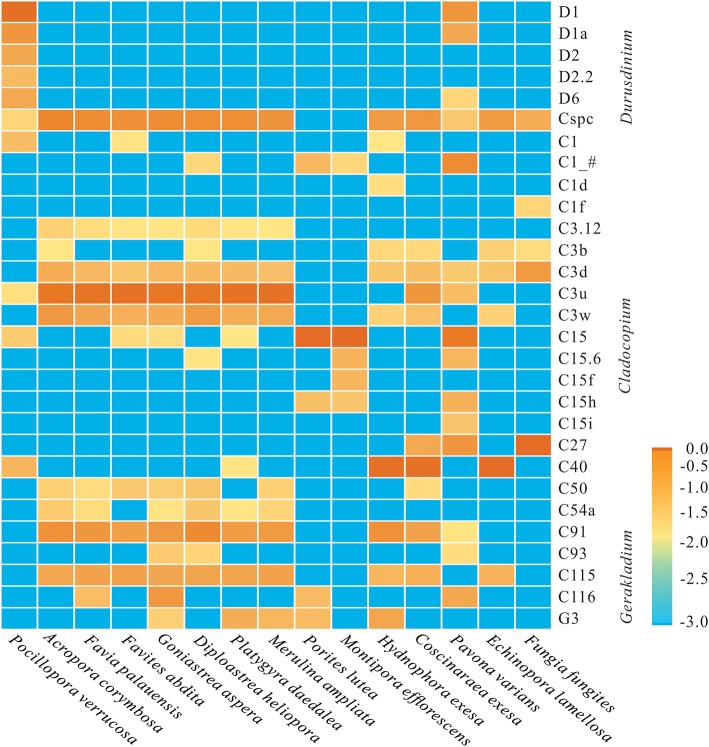
Log-scale percentage heatmap of the dominant/sub-dominant Symbiodiniaceae subclades among 15 coral species. The scales “−3.0, −2.5, −2.0, −1.5, −1.0, −0.5, 0” show the relative abundance at “0, 0.3, 1, 3.1, 10, 31, and 100%,” respectively. The average value was calculated using 3–5 replicates from each coral species.

At the subclade level, based on ITS2 database alignments, a total of 24 dominant/sub-dominant subclades (relative abundances of >1%) were detected, accounting for more than 90% of total sequences (Figure 2). Subclade composition differed significantly among coral species (Wilcoxon rank-sum test, *p* = 0.019). The dominant subclade in *P. verrucosa* was D1 (70.5%), followed by D1a, D2, D6, C40, D2.2, and C1, which accounted for 1–10% of sequences. Dominant subclades in *A. corymbosa* were C3u, Cspc, and C91, with abundances of 44.0, 21.3, and 12.4%, respectively. The dominant/sub-dominant subclades in the five Faviidae coral species (i.e., *F. palauensis, F. abdita, G. aspera, D. heliopora*, and *P. daedalea*) were similar, with all containing C3u, Cspc, C91, C115, C3w, and C3d. *M. ampliata* shared similar dominant subclades with Faviidae. The dominant subclade of *E. lamellosa* (C40 84.8%) was significantly different compared with Faviidae (Wilcoxon rank-sum test, *p* < 0.001). The dominant subclade in *H. exesa* and *C. exesa* was C40 (66.6 and 60.2%, respectively) (Figure 2). In *P. lutea* and *M. efflorescens*, C15 dominated with abundances of 88.9 and 90.0%, respectively, showing high specificity. Finally, the dominant subclade in *F. fungites* was C27 (85.6%), which was clearly different from all other colonial corals in this study.

Shannon diversity index of Symbiodiniaceae indicated that *A. corymbosa*, the five species of Faviidae, *M. ampliata, H. exesa*, and *C. exesa* had relatively high diversity (0.85–1.21), while *P. verrucosa* (0.44), *P. lutea* (0.20), *M. efflorescens* (0.14), and *F. fungites* (0.27) had low diversity (Wilcoxon rank-sum test, *p* = 0.032). This indicates that Symbiodiniaceae assemblages of the former species were higher diversity than the latter group. Although *Cladocopium* was the most represented among the background subclades (with the exception of *P. verrucosa*), each coral species contained multiple subclades belonging to different genera. For example, background types of *A. corymbosa* contained 99 subclades, 94 of which belonged to *Cladocopium*, and five of which belonged to *Durusdinium*. In *P. lutea* and *M. efflorescens*, fewer background subclades were detected (49 and 65, respectively), and more Symbiodiniaceae genera (*Symbiodinium, Cladocopium, Durusdinium, Fugacium, Gerakladium*, and clade I) were found in *P. lutea*. Although most relative abundance of these background subclades was <0.1%, the number and diversity of background subclades among corals showed that they contained different Symbiodiniaceae abundances and diversity (Figures S3, S4; Table S6).

**Figure 3:**
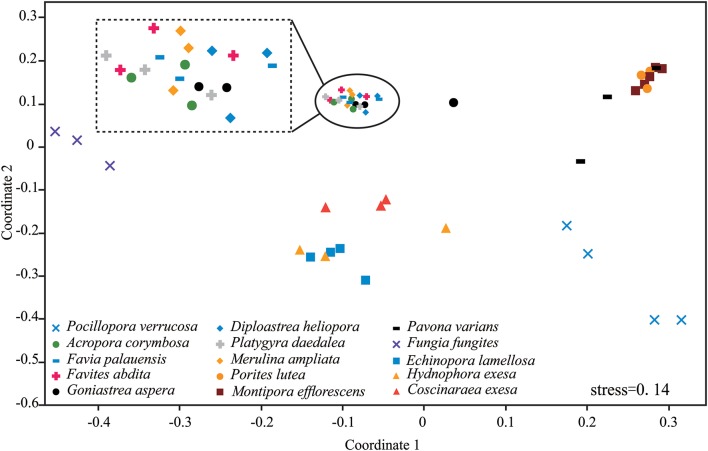
Non-metric multidimensional scaling (nMDS) plotting of the Symbiodiniaceae subclade compositions of the 15 coral species. The 2-D space allows the best spatial representation of sample similarity based on Bray-Curtis similarity indices.

Results of nMDS revealed a clear clustering pattern that varied significantly among coral species (Figure 3). The compositions of *P. verrucosa* and *F. fungites* were extremely different from other corals. *P. lutea, M. efflorescens*, and *P. varians* were similar, but were clearly different from *E. lamellosa, H. exesa*, and *C. exesa*. Symbiodiniaceae subclade compositions were similar among *A. corymbosa, M. ampliata*, and the five species of Faviidae, but clearly different from other coral species.

### The Phylogenetic Relationships Among Symbiodiniaceae Subclades in the 15 Coral Species

**Figure 4:**
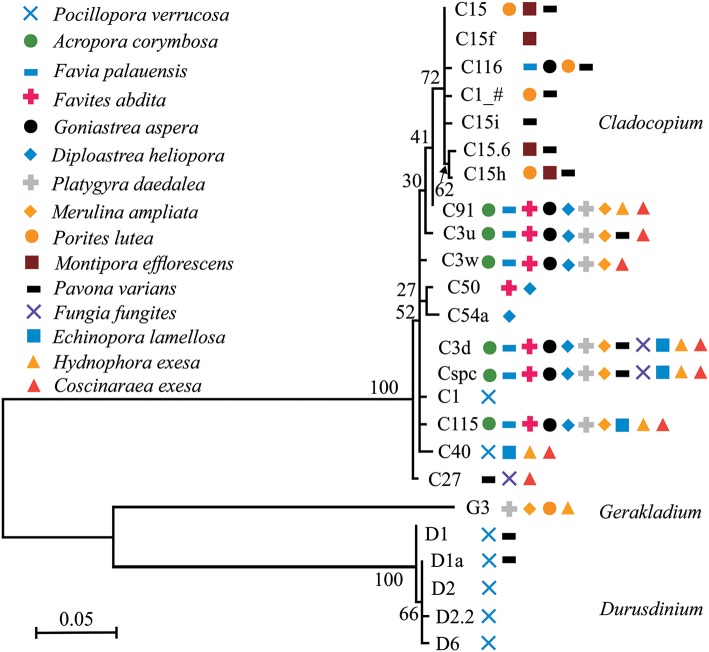
Phylogenetic trees of dominant/sub-dominant Symbiodiniaceae subclades in the 15 coral species. Every symbol represents a group with average relative abundance of a certain subclade >1%. Phylograms were developed based on ITS2 sequences using Maximum Likelihood.

Phylogenetic trees of dominant/sub-dominant Symbiodiniaceae subclades (>1%) and background types (0.1 to 1%) were established (Figure 4; Figures S5–S7). Dominant/sub-dominant Symbiodiniaceae subclades associated with conspecific coral were closely distributed within the phylogenetic trees. For example, D1, D1a, D2, and D2.2 associated with *P. verrucosa* had close phylogenetic relationships. In *A. corymbosa*, phylogenetic relationships between C15, C116, C1_#, and C15h were closer than C91, C3u, C3w, C3d, Cspc, and C115. Phylogenetic relationships between background types had high diversity, but conspecific corals generally had similar relationships. Similar relationships among background types were classified according the phylogenetic tree. The subclades with similar relationships to C15, C3u, C40 were classified as C15-types, C3-types, C40-types, respectively (Figures S6, S7). These results indicate that coral hosts are selectively associated with Symbiodiniaceae dominant/sub-dominant subclades as well as background types, and possibly show coevolution relationships in the symbionts.

## Discussion

### Variation in Zooxanthellae Density in the 15 Coral Species and Their Drivers

In our study, although there were significant differences in ZDs of the 15 coral species, the ZDs of all species were at low levels (0.8–1.2 × 10^6^ cells cm^−2^) compared with those in the northern SCS (Li et al., 2008; Xu et al., 2017). This could be shaped by a variety of environmental factors (e.g., SST, nutrients, water transparency, salinity, or pH). Based on the *in situ* measurement of environmental data, we found that Xinyi Reef is a tropical CRR with high SST and transparency, low turbidity, and oligotrophic conditions. Generally, the main reason for low ZD at tropical reefs is the abnormally high SST that occurs in frequent El Niño under global warming (Hoegh-Guldberg and Salvat, 1995; Hoegh-Guldberg et al., 2007; Li et al., 2011). The third global coral bleaching event occurred from 2015/2016, resulting in severe damage to corals in tropical regions (Hughes et al., 2017). In May 2015, the seawater temperature was 30.4 ± 0.6°C during the sampling period. Corals may be under threat of thermal stress with significantly reduced in zooxanthellae (Fujise et al., 2013; Decarlo et al., 2017). It exhibited lower ZD levels than other reef regions that are below 30°C (Li et al., 2011). In summer, in the Luhuitou fringing reef of northern SCS, for example, ZDs of *Porites, Acropora*, and *Pavona* were ~5 × 10^6^, 2 × 10^6^, and 3 × 10^6^ cells cm^−2^, which were ~4 times, ~2 times, and ~3 times higher than our results, respectively (Xu et al., 2017). As for *Acropora* corals, which have low original levels of ZDs, the densities are lower than other previous reports from low-latitude tropical reefs of the Indo-Pacific, such as French Polynesia (Ladrière et al., 2014) and Kimberley, Australia (Verena et al., 2015). Even for the heat-tolerant *Porites*, Yu and Zhao (2006) found that the corals experienced several severe bleaching disturbances from evidence in the SCS over the past two centuries, suggesting that the main threat to these corals is high SST. Besides, *in situ* ecological observed in the southern SCS showed that 31–90% coral-symbiotic ZDs were lost over all these coral species during abnormally high thermal stress (Li et al., 2011; Yu, 2012).

Nutrients are also important factors influencing the coral-ZDs (Fabricius, 2005; Wiedenmann et al., 2013; Sawall et al., 2014; Ke et al., 2018). In our study, the DIN in this coral reef was 1.38 ± 0.15 μmol L^−1^ and the SRP was 0.06 ± 0.02 μmol L^−1^ during investigation. This indicates that nutrients are in low levels and oligotrophic conditions may limit Symbiodiniaceae reproduction and reduce their population (Ke et al., 2018). Other factors such as solar radiation and water transparency may also affect the ZDs as they directly affect the amount of sunlight available for photosynthesis of Symbiodiniaceae. In the southern SCS, solar radiation can be efficiently transmitted to corals with a high transparency (>20 m) and low turbidity (~0.2 NTU). High light intensity possibly results in the excretion of coral-ZD (Brown, 1997; Hoegh-Guldberg, 1999), resulting in generally low ZD levels.

### Variation in Dominant Symbiodiniaceae Types in Explored Coral Species and Potential Relationships With Coral Adaptability to Thermal Stress

The results from ZDs suggested that there were generally low coral-ZD levels in all coral species in the southern SCS. Although with lower ZDs in the branching *P. verrucosa*, we found that it was the main dominant species and survived well in this CRR. This suggests that ZD may not be the most important factor determining coral thermal adaptability in the southern SCS. In contrast, in our study, there were significant differences in dominant Symbiodiniaceae genera/subclades among these coral species based on the results of the Symbiodiniaceae ITS2 sequence alignments and OTU analysis. These variations of dominant Symbiodiniaceae types may effectively affect corals adaptability to thermal stress.

Recently, the characteristics of heat-tolerance and sensitivity in numerous Symbiodiniaceae at genus/subclade levels have been previously explored (Kemp et al., 2014; Silverstein et al., 2015; Swain et al., 2017). This contributed to our rationale for investigating coral adaptability to thermal stress among species. In the Indo-Pacific, many symbiotic subclades are considered to be heat-sensitive (e.g., C3, C7, and A13), while some subclades in *Cladocopium* (e.g., C15, C15-like types) and *Durusdinium* (e.g., D1, D1–4 and D1a) are considered to be heat-tolerant (e.g., Hume et al., 2015; Silverstein et al., 2015; Swain et al., 2017). Long-term tropical environmental exposure has forced corals to become associated with dominant heat-tolerant Symbiodiniaceae, giving them higher resistance to thermal stress (Kennedy et al., 2015; Hume et al., 2016; Brener-Raffalli et al., 2018). Generally, corals associated with *Durusdinium* can enhance their tolerance to thermal stress (Berkelmans and Oppen, 2006; Kennedy et al., 2015). For instance, Tong et al. (2017) found that the symbiotic dominant Symbiodiniaceae of *Galaxea fascicularis* belong to *Durusdinium*, which is significantly more resistant to thermal stress than the *Cladocopium* members associated with *Montipora* corals. In our study, *P. verrucosa* was associated with dominant *Durusdinium* of D1 and D1a, and *P. lutea* and *M. efflorescens* were associated with dominant C15. These Symbiodiniaceae types are considered to be highly adaptable to thermal stress (Hume et al., 2015; Silverstein et al., 2015; Swain et al., 2017). Based on coral evolutionary relationships (Figure S8), corals with closer phylogenetic relationships seem to associate with similar dominant Symbiodiniaceae types. This suggests that the choice of dominant subclade is mainly controlled by the coral host (Sampayo et al., 2008; Putnam et al., 2012; Klepac et al., 2015). Therefore, theoretically, corals associated with heat-tolerant Symbiodiniaceae may have high thermal tolerance.

The cause of coral species (e.g., *P. verrucosa*) becoming dominant can be understood by the variations in dominant Symbiodiniaceae types in the southern SCS. Our study suggests that the main reason of the branching *P. verrucosa* corals becoming dominant and surviving under thermal stress conditions is that they are associated with heat-tolerant *Durusdinium*. Similarly, the *P. lutea* and *M. efflorescens* associated with C15 may also have relatively high thermal tolerance and become dominant in the southern SCS.

### Diversity of Background Symbiodiniaceae Types and Potential Relationship With Coral Adaptability to Future Global Warming

Symbiodiniaceae background types were significantly different among corals regardless of subclade types or diversity. The subclades in corals such as *P. verrucosa* and *A. corymbosa* were relatively highly diverse, but this was much lower in *P. lutea, M. efflorescens*, and *F. fungites*. In tropical reefs, corals may improve their adaptability to climate change by increasing the diversity of symbiotic Symbiodiniaceae. General corals such as *P. verrucosa* could increase their diversity by developing symbiotic relationships with different free-living Symbiodiniaceae (Baker, 2003; Putnam et al., 2012; Fabina et al., 2013). Horizontal transmission patterns are beneficial for these general corals to develop associations with various Symbiodiniaceae, including heat-tolerant types (Boulotte et al., 2016; Quigley et al., 2017). A rapid breeding strategy allows corals to obtain more heat-tolerant Symbiodiniaceae from the surrounding water and to increase survival in the short term (Putnam et al., 2012; Smith et al., 2017a). In our study, the dominant Symbiodiniaceae in *P. verrucosa* are heat-tolerant *Durusdinium* (e.g., D1 and D1a), and its background Symbiodiniaceae assemblage also include numerous heat-tolerant types, indicating that this species adapts by associating with a high proportion of heat-tolerant Symbiodiniaceae. Corals with high diversity can adapt to thermal conditions by altering the abundance of heat-tolerant background Symbiodiniaceae. *A. corymbosa, F. palauensis, F. abdita, G. aspera, D. heliopora*, and *P. daedalea* also belong to general corals and have numerous associated background types. Although their dominant subclades are *Cladocopium* C3u and Cspc, a variety of heat-tolerant background types (e.g., D1, C40, C15, and C15.6) were also detected, indicating high symbiotic diversity. These coral species include numerous Symbiodiniaceae including *Symbiodinium, Breviolum, Cladocopium, Durusdinium, Fugacium*, and *Gerakladium*. High diversity of Symbiodiniaceae may help these corals survive under thermal stress, despite not being dominant in the southern SCS.

In addition, previous studies have found that *P. lutea* and C15 are closely associated in the northern SCS and have observed a coevolution between the two (Ng and Ang, 2016; Gong et al., 2018). Consistently, we found that C15 was also the dominant Symbiodiniaceae subclade of *P. lutea* in the southern SCS. However, we additionally detected 49 background subclades, including a variety from *Symbiodinium, Breviolum, Cladocopium, Durusdinium, Fugacium*, and *Gerakladium*, implying that even coral species with high symbiont specificity are associated with diverse and adaptable background assemblages. These results indicate that *P. lutea* in the southern SCS can possibly adapt to high temperature environments and/or recover from bleaching events via relatively high symbiosis diversity under future global warming. Overall, our results provide an important basis for better understanding the coral-Symbiodiniaceae community population, composition, and diversity among coral species in the southern SCS and benefit for insight into variation in thermal adaptability among corals in field.

## Data Availability Statement

The data used in this paper can be obtained from Supplementary Materials or requested by emailing KY at kefuyu@scsio.ac.cn.

## Author Contributions

ZQ and KY conceived and designed the experiments and wrote the manuscript. ZQ and BC performed the experiments and analyzed the experimental data. ZQ, YW, JL, WL, LX, and XH contributed to reagents and materials. All authors edited and approved the manuscript.

### Conflict of Interest

The authors declare that the research was conducted in the absence of any commercial or financial relationships that could be construed as a potential conflict of interest.
