# Supplementary material for: Diversity of Symbiodiniaceae in 15 Coral Species From the Southern South China Sea: Potential Relationship With Coral Thermal Adaptability
Source: Front Microbiol. 2019 Oct 18;10:2343. doi: 10.3389/fmicb.2019.02343 (PMC6813740; doi:10.3389/fmicb.2019.02343)
Supplement: Supplementary file 1 [file Table_1.DOCX]

**Supplementary Materials for:**

# Diversity of Symbiodiniaceae in fifteen coral species from the southern South China Sea: Potential relationship with coral thermal adaptability

**Zhenjun Qin^1^**^–^**^4^, Kefu Yu^1^**^–^**^3*^, Biao Chen^1^**^–^**^3^, Yinghui Wang^1^**^–^**^3^, Jiayuan Liang^1^**^–^**^3^, Wenwen Luo^1^**^–^**^4^, Lijia Xu^1^**^–^**^3^, Xueyong Huang^1^**^–^**^4^**

^1^Coral Reef Research Center of China, Guangxi University, Nanning, 530004, China

^2^Guangxi Laboratory on the Study of Coral Reefs in the South China Sea, Nanning, 530004, China

^3^School of Marine Sciences, Guangxi University, Nanning, 530004, China

^4^College of Forestry, Guangxi University, Nanning, 530004, China

**Corresponding author:** Dr. Kefu Yu

E-mail: kefuyu@scsio.ac.cn

Tel: +86-771-3231358

**Contents of this file**

Tables S1 to S5

**Additional Supporting Information**

Captions for Table S6, Figures S1 to S8 and ITS2 Database

**Table S1** The coordinates, sampling time, and sampling depth and coral genera/species identification in the Xinyi Reef, southern South China Sea.

| Site | longitude | latitude | Sampling time (m-d-y) | Depth (m) | Genus | Species | Number of samples |
| --- | --- | --- | --- | --- | --- | --- | --- |
| Xinyi Reef, southern SCS | 115°55′49″E | 9°20′6″N | 06-08-2015 | 6~10 | *Pocillopora* | *P. verrucosa* | 4 |
|  |  |  | 06-08-2015 | 6~10 | *Acropora* | *A. corymbosa* | 3 |
|  |  |  | 06-08-2015 | 6~10 | *Favia* | *F. palauensis* | 3 |
|  |  |  | 06-08-2015 | 6~10 | *Favites* | *F. abdita* | 3 |
|  |  |  | 06-08-2015 | 6~10 | *Goniastrea* | *G. aspera* | 3 |
|  |  |  | 06-08-2015 | 6~10 | *Diploastrea* | *D. heliopora* | 3 |
|  |  |  | 06-08-2015 | 6~10 | *Platygyra* | *P. daedalea* | 3 |
|  |  |  | 06-08-2015 | 6~10 | *Porites* | *P. lutea* | 3 |
|  |  |  | 06-08-2015 | 6~10 | *Merulina* | *M. ampliata* | 5 |
|  |  |  | 06-08-2015 | 6~10 | *Montipora* | *M. efflorescens* | 3 |
|  |  |  | 06-08-2015 | 6~10 | *Hydnophora* | *H. exesa* | 3 |
|  |  |  | 06-08-2015 | 6~10 | *Coscinaraea* | *C. exesa* | 3 |
|  |  |  | 06-08-2015 | 6~10 | *Pavona* | *P. varians* | 3 |
|  |  |  | 06-08-2015 | 6~10 | *Echinopora* | *E. lamellosa* | 3 |
|  |  |  | 06-08-2015 | 6~10 | *Fungia* | *F. fungites* | 3 |

**Table S2** The mean annual sea surface temperature (SST, °C) for Xinyi Reef, southern SCS (2015, mean ± sd).

| Site | Spring | Summer | Autumn | Winter | Mean SST |
| --- | --- | --- | --- | --- | --- |
| Xinyi Reef | 28.1 ± 0.7 | 30.4 ± 1.3 | 29.2 ± 0.6 | 28.6 ± 0.5 | 29.1 ± 2.6 |

**Table S3** Environmental data during sampling period (mean ± sd).

| Site | SST (°C) | Depth (m) | Salinity (‰) | DO (mg/L) | DIN (μmol L^-1^) | SRP (μmol L^-1^) | Transparency (m) | Turbidity (NTU) | pH |
| --- | --- | --- | --- | --- | --- | --- | --- | --- | --- |
| Xinyi Reef | 31.1 ± 0.6 | 6 ± 0.5 | 33.1 ± 0.8 | 6.85 ± 0.04 | 1.38 ± 0.15 | 0.06 ± 0.02 | 24.3 ± 2.5 | 0.2 ± 0.03 | 8.24 ± 0.14 |

**Table S4** The zooxanthellae density of the explored corals in Xinyi Reef, the Spratly Islands.

| Corals | zooxanthellae density (×10^6^ cells cm^-2^) | | | | | | | | |
| --- | --- | --- | --- | --- | --- | --- | --- | --- | --- |
|  | 1 | 2 | 3 | 4 | 5 | 6 | 7 | 8 | Mean ± sd |
| Poc_1 | 0.88 | 0.90 | 0.94 | 0.84 | 0.80 | 0.90 | 0.88 | 0.88 | 0.86 ± 0.12 |
| Poc_2 | 0.66 | 0.60 | 0.68 | 0.76 | 0.72 | 0.70 | 0.78 | 0.76 |  |
| Poc_3 | 1.08 | 1.16 | 1.10 | 1.00 | 0.96 | 0.88 | 0.92 | 1.00 |  |
| Poc_4 | 0.82 | 0.84 | 0.86 | 0.84 | 0.88 | 0.82 | 0.86 | 0.80 |  |
| Acr_1 | 0.82 | 0.84 | 0.84 | 0.80 | 0.86 | 0.88 | 0.80 | 0.76 | 0.84 ± 0.09 |
| Acr_2 | 0.92 | 1.00 | 1.08 | 0.90 | 0.96 | 0.88 | 0.90 | 0.86 |  |
| Acr_3 | 0.82 | 0.76 | 0.72 | 0.80 | 0.64 | 0.76 | 0.82 | 0.76 |  |
| Fav_1 | 1.16 | 1.20 | 1.20 | 1.22 | 1.18 | 1.20 | 1.24 | 1.20 | 1.20 ± 0.08 |
| Fav_2 | 1.06 | 1.14 | 1.16 | 1.08 | 1.10 | 1.12 | 1.16 | 1.14 |  |
| Fav_3 | 1.30 | 1.36 | 1.32 | 1.28 | 1.30 | 1.24 | 1.26 | 1.24 |  |
| Fat_1 | 0.90 | 1.00 | 1.04 | 1.12 | 1.10 | 1.16 | 1.18 | 1.20 | 1.21 ± 0.13 |
| Fat_2 | 1.24 | 1.40 | 1.36 | 1.30 | 1.30 | 1.48 | 1.32 | 1.34 |  |
| Fat_3 | 1.22 | 1.22 | 1.20 | 1.24 | 1.20 | 1.16 | 1.26 | 1.20 |  |
| Gon_1 | 1.34 | 1.30 | 1.28 | 1.26 | 1.30 | 1.28 | 1.26 | 1.20 | 1.22 ± 0.08 |
| Gon_2 | 1.10 | 1.04 | 1.12 | 1.16 | 1.20 | 1.24 | 1.10 | 1.12 |  |
| Gon_3 | 1.26 | 1.26 | 1.16 | 1.24 | 1.22 | 1.28 | 1.24 | 1.20 |  |
| Dip_1 | 0.98 | 0.96 | 1.00 | 0.98 | 0.96 | 0.98 | 1.02 | 1.00 | 0.99 ± 0.11 |
| Dip_2 | 1.22 | 1.16 | 1.12 | 1.10 | 1.04 | 1.06 | 1.12 | 1.08 |  |
| Dip_3 | 0.90 | 0.80 | 0.84 | 0.74 | 0.86 | 0.92 | 0.96 | 0.96 |  |
| Pla_1 | 0.90 | 0.86 | 0.88 | 0.92 | 0.96 | 0.94 | 0.90 | 0.98 | 0.98 ± 0.07 |
| Pla_2 | 1.08 | 1.10 | 1.06 | 1.04 | 1.08 | 1.04 | 0.98 | 0.98 |  |
| Pla_3 | 0.98 | 0.96 | 0.98 | 1.00 | 0.96 | 0.94 | 1.04 | 1.06 |  |
| Mer_1 | 0.90 | 0.90 | 0.92 | 0.92 | 0.94 | 0.86 | 0.88 | 0.80 | 0.89 ± 0.12 |
| Mer_2 | 0.78 | 0.80 | 0.84 | 0.86 | 0.72 | 0.70 | 0.76 | 0.68 |  |
| Mer_3 | 1.00 | 1.04 | 1.00 | 1.06 | 1.04 | 1.08 | 0.96 | 0.94 |  |
| Por_1 | 1.18 | 1.24 | 1.20 | 1.30 | 1.20 | 1.24 | 1.16 | 1.18 | 1.18 ± 0.07 |
| Por_2 | 1.04 | 1.02 | 1.10 | 1.12 | 1.10 | 1.14 | 1.16 | 1.16 |  |
| Por_3 | 1.24 | 1.24 | 1.20 | 1.16 | 1.20 | 1.18 | 1.18 | 1.30 |  |
| Mon_1 | 0.98 | 1.00 | 0.98 | 1.04 | 1.08 | 1.06 | 1.04 | 1.04 | 1.16 ± 0.09 |
| Mon_2 | 1.16 | 1.16 | 1.18 | 1.20 | 1.14 | 1.16 | 1.16 | 1.18 |  |
| Mon_3 | 1.24 | 1.32 | 1.28 | 1.38 | 1.20 | 1.26 | 1.28 | 1.24 |  |
| Mon_4 | 1.12 | 1.10 | 1.16 | 1.12 | 1.14 | 1.18 | 1.20 | 1.16 |  |
| Mon_5 | 1.14 | 1.20 | 1.22 | 1.20 | 1.18 | 1.16 | 1.24 | 1.12 |  |
| Hyd_1 | 1.08 | 1.12 | 1.10 | 1.14 | 1.18 | 1.06 | 1.02 | 1.00 | 1.00 ± 0.10 |
| Hyd_2 | 0.84 | 0.90 | 0.88 | 0.82 | 0.92 | 0.86 | 0.90 | 0.94 |  |
| Hyd_3 | 1.06 | 1.04 | 1.00 | 1.00 | 1.02 | 1.06 | 1.04 | 1.08 |  |
| Cos_1 | 0.98 | 0.98 | 0.96 | 0.96 | 0.96 | 0.94 | 0.94 | 0.90 | 0.97 ± 0.07 |
| Cos_2 | 1.06 | 1.00 | 1.04 | 1.06 | 1.08 | 1.10 | 1.04 | 1.02 |  |
| Cos_3 | 0.80 | 0.84 | 0.90 | 0.94 | 0.92 | 0.96 | 0.94 | 0.90 |  |
| Pav_1 | 0.86 | 0.86 | 0.90 | 0.84 | 0.88 | 0.88 | 0.90 | 0.88 | 0.88 ± 0.08 |
| Pav_2 | 1.06 | 1.00 | 0.96 | 0.98 | 0.94 | 0.92 | 0.90 | 0.90 |  |
| Pav_3 | 0.80 | 0.76 | 0.78 | 0.72 | 0.84 | 0.86 | 0.88 | 0.82 |  |
| Ech_1 | 1.08 | 1.04 | 1.02 | 1.06 | 1.04 | 1.08 | 1.02 | 0.98 | 0.94 ± 0.10 |
| Ech_2 | 0.82 | 0.86 | 0.90 | 0.94 | 0.92 | 0.96 | 0.94 | 1.02 |  |
| Ech_3 | 0.96 | 0.76 | 0.84 | 0.90 | 0.74 | 0.92 | 0.82 | 0.90 |  |
| Fun_1 | 1.02 | 1.06 | 1.14 | 1.00 | 0.96 | 0.94 | 0.90 | 0.92 | 0.84 ± 0.13 |
| Fun_2 | 0.84 | 0.86 | 0.84 | 0.84 | 0.86 | 0.82 | 0.80 | 0.76 |  |
| Fun_3 | 0.80 | 0.72 | 0.70 | 0.68 | 0.64 | 0.70 | 0.64 | 0.80 |  |

**Table S5** Summary of next generation sequencing data and Shannon diversity index. The table includes sampled corals, mean, min and max length of ITS2 sequences, number of Symbiodiniaceae ITS2 sequences and Shannon diversity indices (H’).

| Corals | Mean length | Min length | Max length | Number of Symbiodiniaceae ITS2 sequences | Shannon (H’) |
| --- | --- | --- | --- | --- | --- |
| Poc_1 | 313.04 | 279 | 391 | 37285 | 0.0945 |
| Poc_2 | 312.69 | 279 | 327 | 47679 | 1.2932 |
| Poc_3 | 310.03 | 277 | 337 | 30083 | 0.0896 |
| Poc_4 | 307.19 | 257 | 326 | 39668 | 0.2987 |
| Acr_1 | 314.61 | 297 | 365 | 52583 | 1.0808 |
| Acr_2 | 314.46 | 279 | 365 | 44454 | 1.2074 |
| Acr_3 | 314.66 | 295 | 365 | 31914 | 0.9432 |
| Fav_1 | 314.63 | 237 | 404 | 37098 | 1.0388 |
| Fav_2 | 314.65 | 253 | 365 | 48240 | 0.9206 |
| Fav_3 | 314.64 | 253 | 404 | 37532 | 1.0607 |
| Fat_1 | 313.94 | 252 | 329 | 50243 | 0.5566 |
| Fat_2 | 314.78 | 254 | 446 | 32573 | 1.037 |
| Fat_3 | 314.87 | 276 | 343 | 51780 | 0.9727 |
| Gon_1 | 315 | 254 | 376 | 34370 | 1.1376 |
| Gon_2 | 314.41 | 241 | 447 | 42730 | 1.186 |
| Gon_3 | 314.86 | 254 | 400 | 43597 | 1.149 |
| Dip_1 | 314.59 | 253 | 319 | 51506 | 1.101 |
| Dip_2 | 314.88 | 256 | 404 | 39288 | 1.3489 |
| Dip_3 | 314.63 | 253 | 404 | 62215 | 0.9757 |
| Pla_1 | 314.79 | 298 | 350 | 37709 | 0.765 |
| Pla_2 | 316.73 | 287 | 361 | 37283 | 1.3591 |
| Pla_3 | 314.63 | 261 | 391 | 52854 | 1.019 |
| Mer_1 | 315.6 | 286 | 424 | 61848 | 1.1108 |
| Mer_2 | 314.65 | 243 | 342 | 35826 | 0.8613 |
| Mer_3 | 314.68 | 253 | 365 | 62678 | 0.8249 |
| Por_1 | 316.06 | 254 | 359 | 49210 | 0.489 |
| Por_2 | 314.95 | 296 | 375 | 48552 | 0.064 |
| Por_3 | 314.98 | 301 | 349 | 39926 | 0.0534 |
| Mon_1 | 314.83 | 222 | 343 | 39492 | 0.1946 |
| Mon_2 | 314.57 | 221 | 341 | 43658 | 0.098 |
| Mon_3 | 314.56 | 279 | 383 | 33935 | 0.1194 |
| Mon_4 | 314.91 | 297 | 319 | 31017 | 0.2124 |
| Mon_5 | 314.79 | 233 | 334 | 37061 | 0.0626 |
| Hyd_1 | 317.01 | 293 | 353 | 43535 | 1.1128 |
| Hyd_2 | 314.82 | 240 | 372 | 60030 | 0.702 |
| Hyd_3 | 315.68 | 295 | 341 | 38732 | 1.3371 |
| Cos_1 | 314.95 | 253 | 351 | 36890 | 1.1158 |
| Cos_2 | 314.76 | 276 | 335 | 45234 | 1.2426 |
| Cos_3 | 313.96 | 253 | 404 | 64358 | 1.2756 |
| Pav_1 | 314.93 | 286 | 355 | 41084 | 0.0502 |
| Pav_2 | 311.91 | 257 | 427 | 50006 | 1.1604 |
| Pav_3 | 314.84 | 254 | 368 | 52812 | 0.4478 |
| Ech_1 | 314.8 | 275 | 349 | 32214 | 0.6385 |
| Ech_2 | 314.89 | 293 | 350 | 37458 | 0.6671 |
| Ech_3 | 314.82 | 266 | 346 | 56788 | 0.6352 |
| Fun_1 | 309.16 | 296 | 317 | 40445 | 0.2446 |
| Fun_2 | 309.2 | 294 | 330 | 63558 | 0.276 |
| Fun_3 | 309.18 | 292 | 318 | 53261 | 0.3033 |

**Table S6** The types of Symbiodiniaceae subclades in the explored coral species in Xinyi Reef, the Spratly Islands.

**Supplementary material ITS2 Database.** ITS2 Database was available under Arif et al. (2014), Chen et al. (2019), and Franklin et al. (2012).

**Figure S1** Map of study area. Sampling area is indicated by the red box and circle.

**Figure S2** Sea surface temperatures (SSTs) at the study site of Xinyi Reef, South China Sea. These were obtained from NASA satellite-derived datasets based on ocean color radiometry via monthly averaged MODIS-Aqua 9 km from January 1997 to December 2015 (<http://oceandata.sci.gsfc.nasa.gov/>).

**Figure S3** Background subclades (relative abundance <1%) among coral species. The inner circle represents dominant/subdominant subclades, and background subclades are represented as BAST (background abundances of Symbiodiniaceae types) in the circle. The outer ring represents relative abundance of background subclades. Background types of relative abundance 0.1–1% are shown on the map. And the relative abundance of subclades <0.1% are classified as “others”.

**Figure S4** Background subclades (relative abundance <1%) among coral species (Figure S3 continued).

**Figure S5** Phylogenetic analysis of the dominant Symbiodiniaceae in the fifteen coral species in the southern SCS based on Bayesian inference.

**Figure S6** Phylogenetic tree of background Symbiodiniaceae subclades (0.1%~1%) in the fifteen coral species. Every symbol represents a group in which average relative abundance of a certain subclade is 0.1~1%. Phylograms are analyzed with Symbiodiniaceae ITS2 sequences using maximum likelihood analyses.

**Figure S7** Phylogenetic analysis of the background Symbiodiniaceae (0.1%~1%) in the fifteen coral species in the southern SCS based on Bayesian inference.

**Figure S8** Scleractinian corals mapped onto the phylogeny, modified from Huang et al. (2014), Kerr (2005), and Swan et al. (2017).

Reference

Arif, C., Daniels, C., Bayer, T., Banguerahinestroza, E., Barbrook, A., Howe, C. J., Lajeunesse, T. C., and Voolstra, C. R. (2014). Assessing *Symbiodinium* diversity in scleractinian corals via next-generation sequencing-based genotyping of the ITS2 rDNA region. *Mol. Ecol.* 23:4418–4433. https://doi.org/10.1111/mec.12869

Chen, B., Yu, K., Liang, J., Huang, W., Wang, G., Su, H., Qin, Z., Huang, X., et al. (2019). Latitudinal variation in the molecular diversity and community composition of Symbiodiniaceae in coral from the South China Sea. *Front. Microbiol.* 10:1278. doi: 10.3389/fmicb.2019.01278

Franklin, E. C., Stat, M., Pochon, X., Putnam, H. M., and Gates, R. D. (2012). GeoSymbio: a hybrid, cloud‐based web application of global geospatial bioinformatics and ecoinformatics for *Symbiodinium*-host symbioses. *Mol. Ecol. Resour.* 12:369–373. https://doi.org/10.1111/j.1755-0998.2011.03081.x

Huang, D., Meier, R., Todd, P. A., and Chou, L. M. (2009). More evidence for pervasive paraphyly in scleractinian corals: systematic study of Southeast Asian Faviidae (Cnidaria; Scleractinia) based on molecular and morphological data. *Mol. Phylogenet. Evol.* 50:102–116. https://doi.org/10.1016/j.ympev.2008.10.012

Kerr, A. M. (2005). Molecular and morphological supertree of stony corals (Anthozoa: Scleractinia) using matrix representation parsimony. *Biological Reviews of the Cambridge Philosophical Society* 80:543. https://doi.org/10.1017/s1464793105006780

Swain, T. D., Chandler, J., Backman, V., and Marcelino, L. (2017). Consensus thermotolerance ranking for 110 *Symbiodinium* phylotypes: an exemplar utilization of a novel iterative partial‐rank aggregation tool with broad application potential. *Funct. Ecol.* 31. https://doi.org/10.1111/1365-2435.12694
