# Supplementary figures and images for: Diversity of Symbiodiniaceae in 15 Coral Species From the Southern South China Sea: Potential Relationship With Coral Thermal Adaptability
Source: Front Microbiol. 2019 Oct 18;10:2343. doi: 10.3389/fmicb.2019.02343 (PMC6813740; doi:10.3389/fmicb.2019.02343)

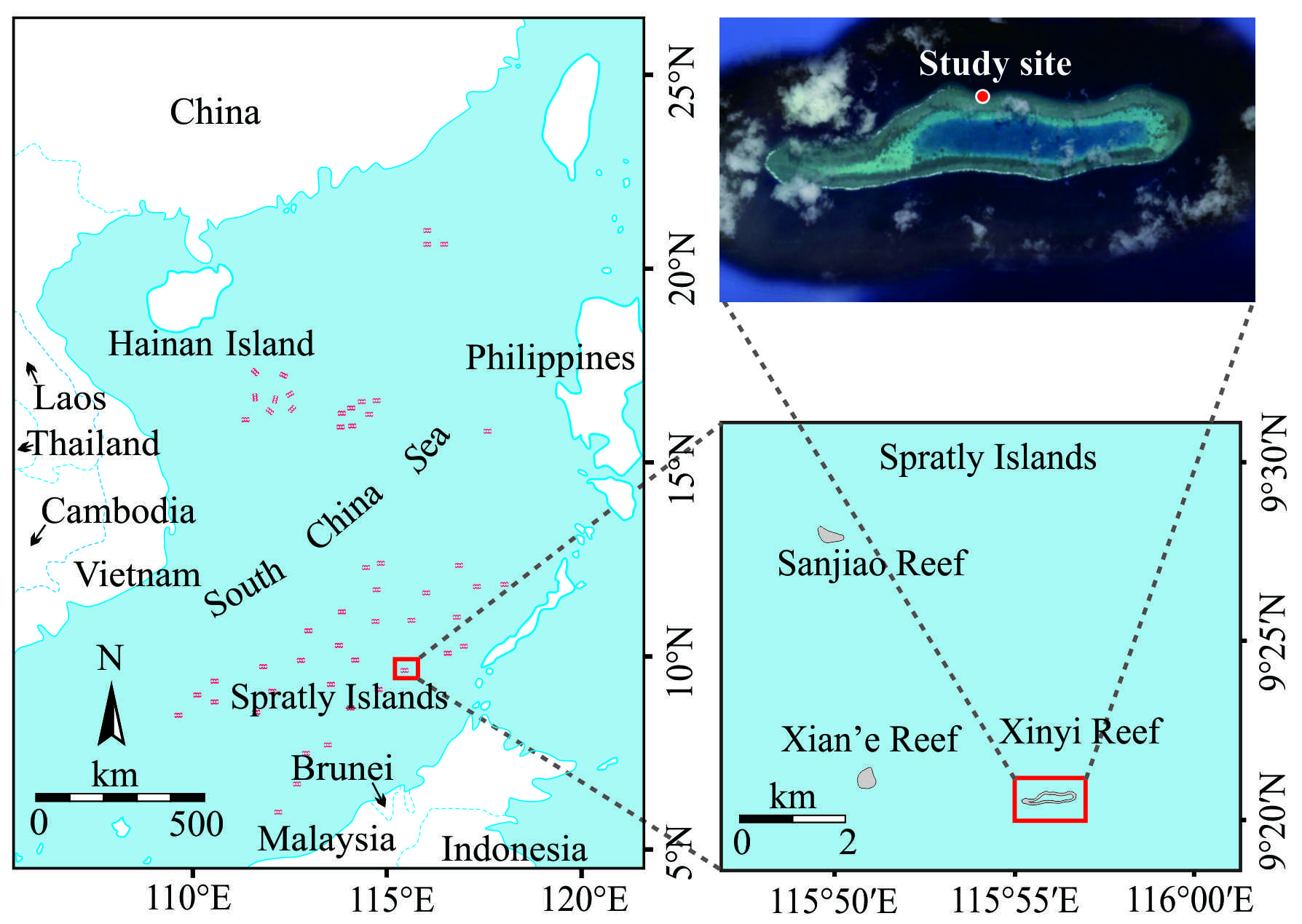

Supplement: Supplementary file 4 [file Image_1.JPEG]

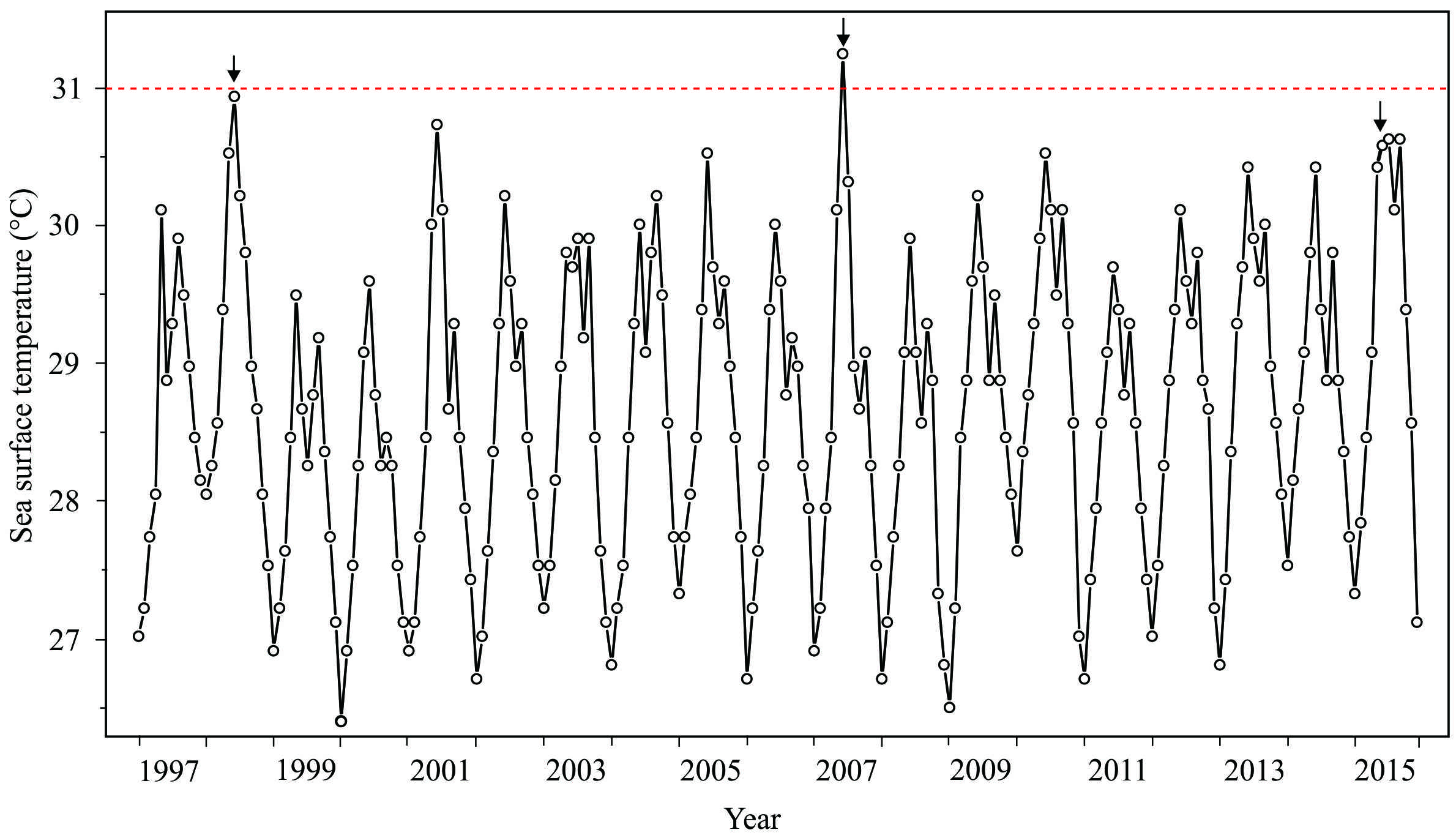

Supplement: Supplementary file 5 [file Image_2.JPEG]

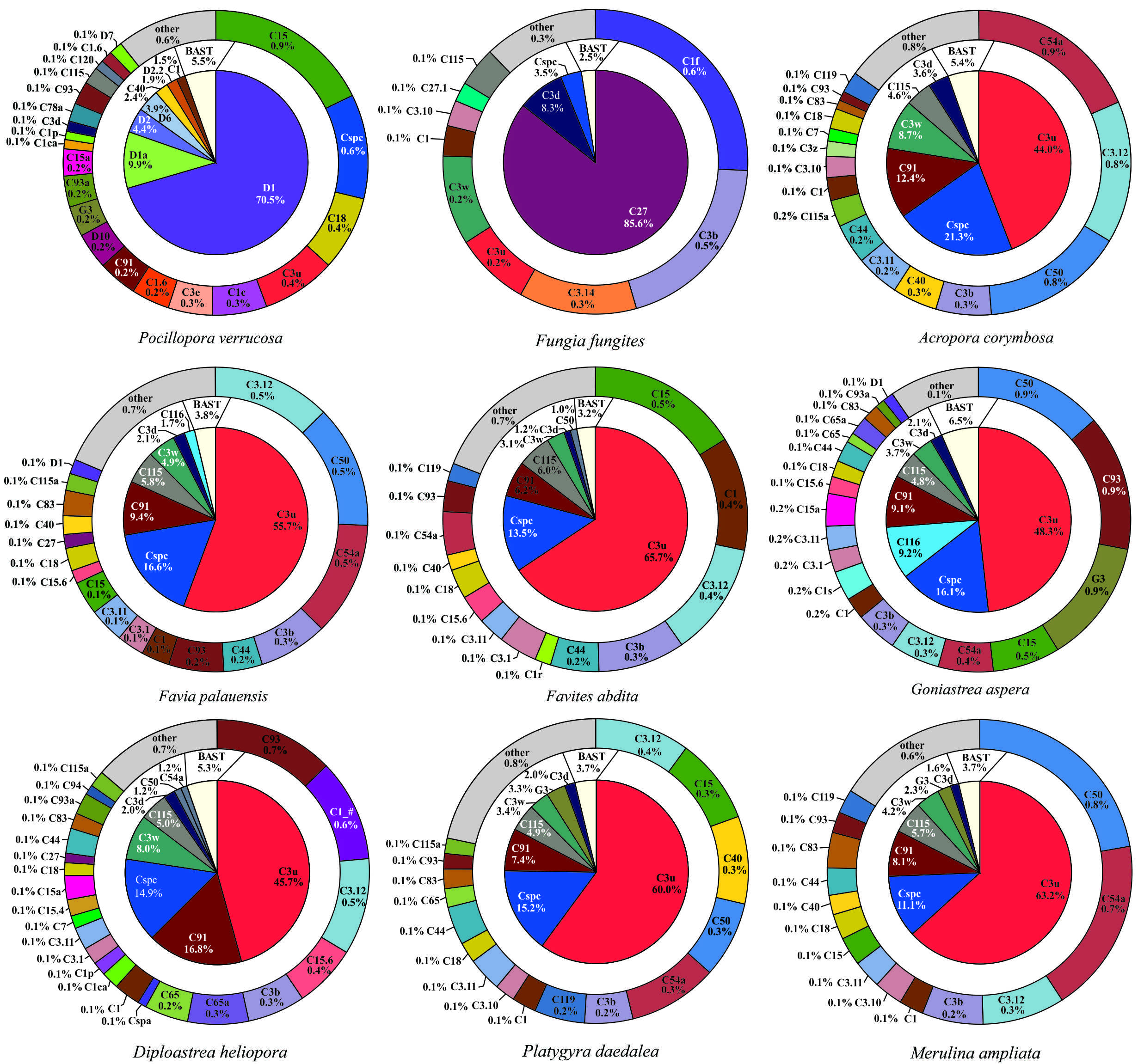

Supplement: Supplementary file 6 [file Image_3.JPEG]

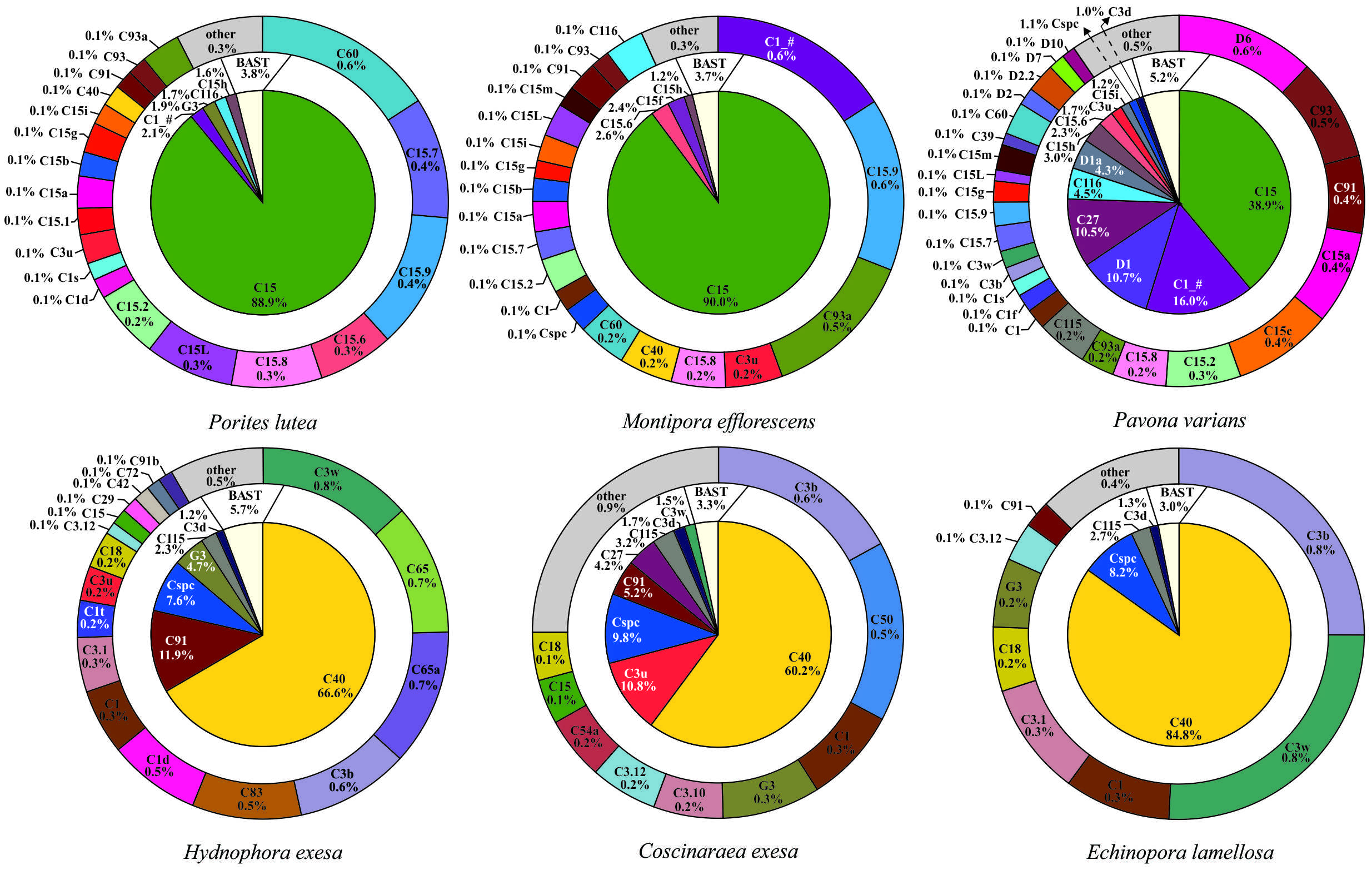

Supplement: Supplementary file 7 [file Image_4.JPEG]

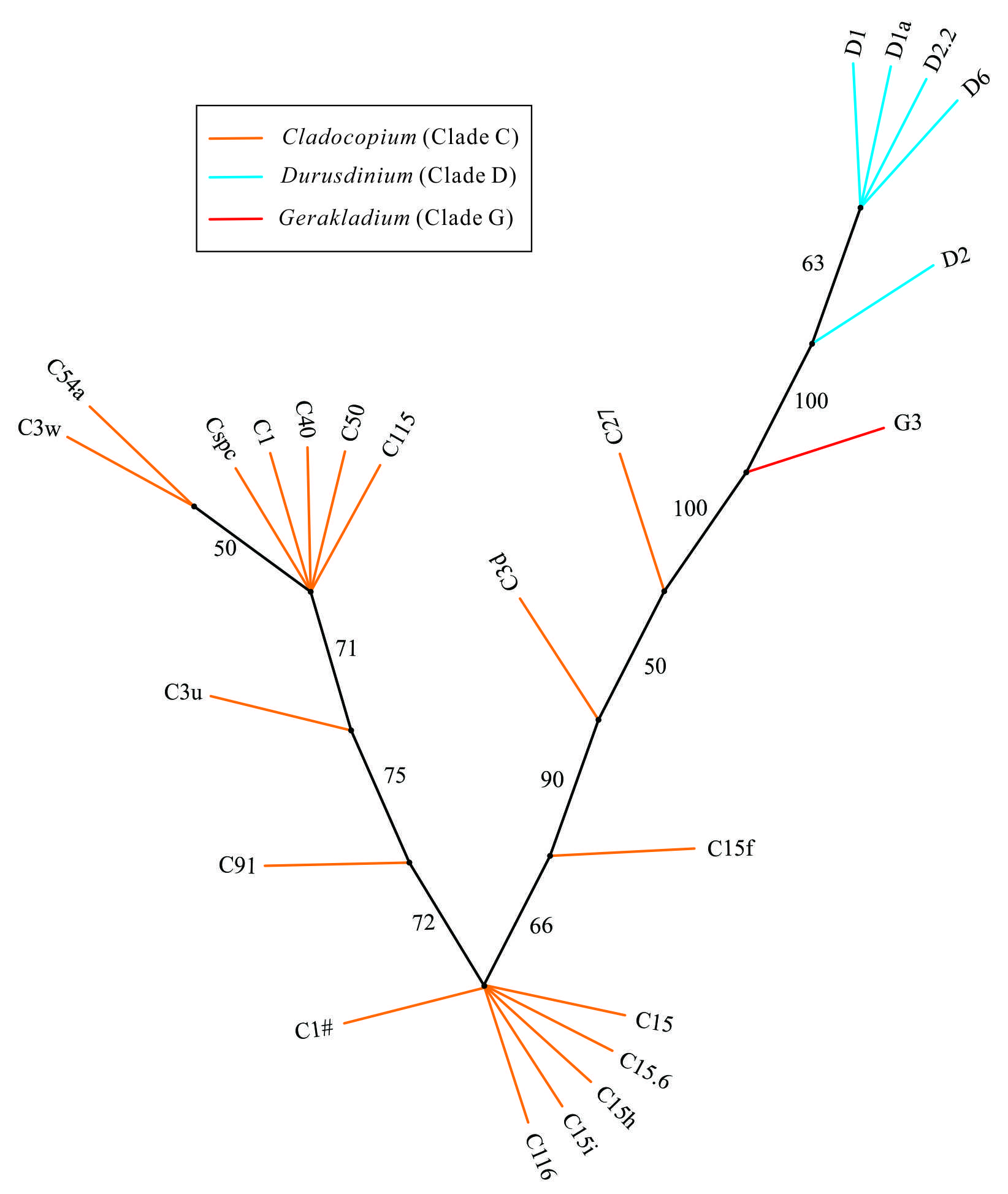

Supplement: Supplementary file 8 [file Image_5.JPEG]

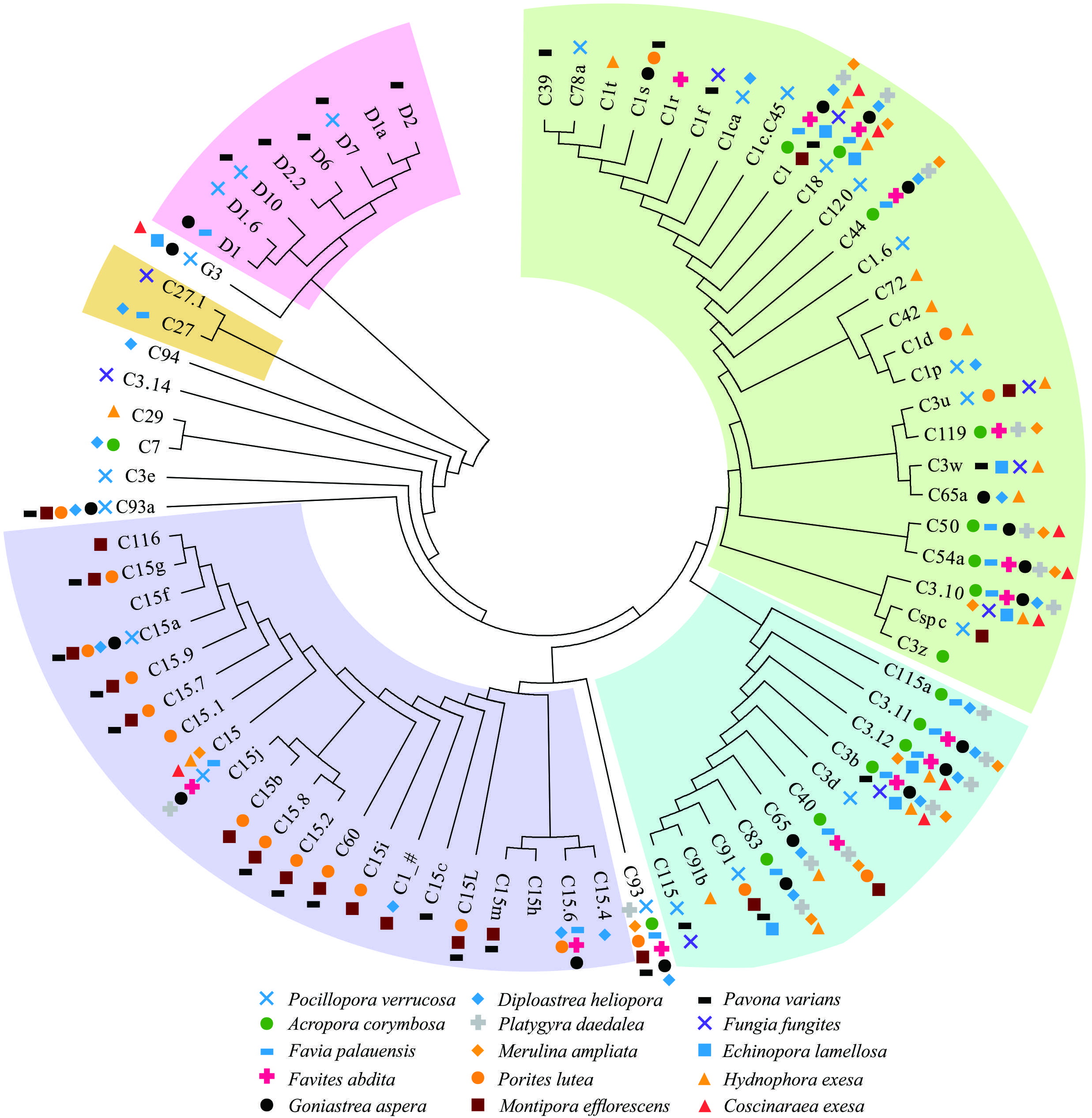

Supplement: Supplementary file 9 [file Image_6.JPEG]

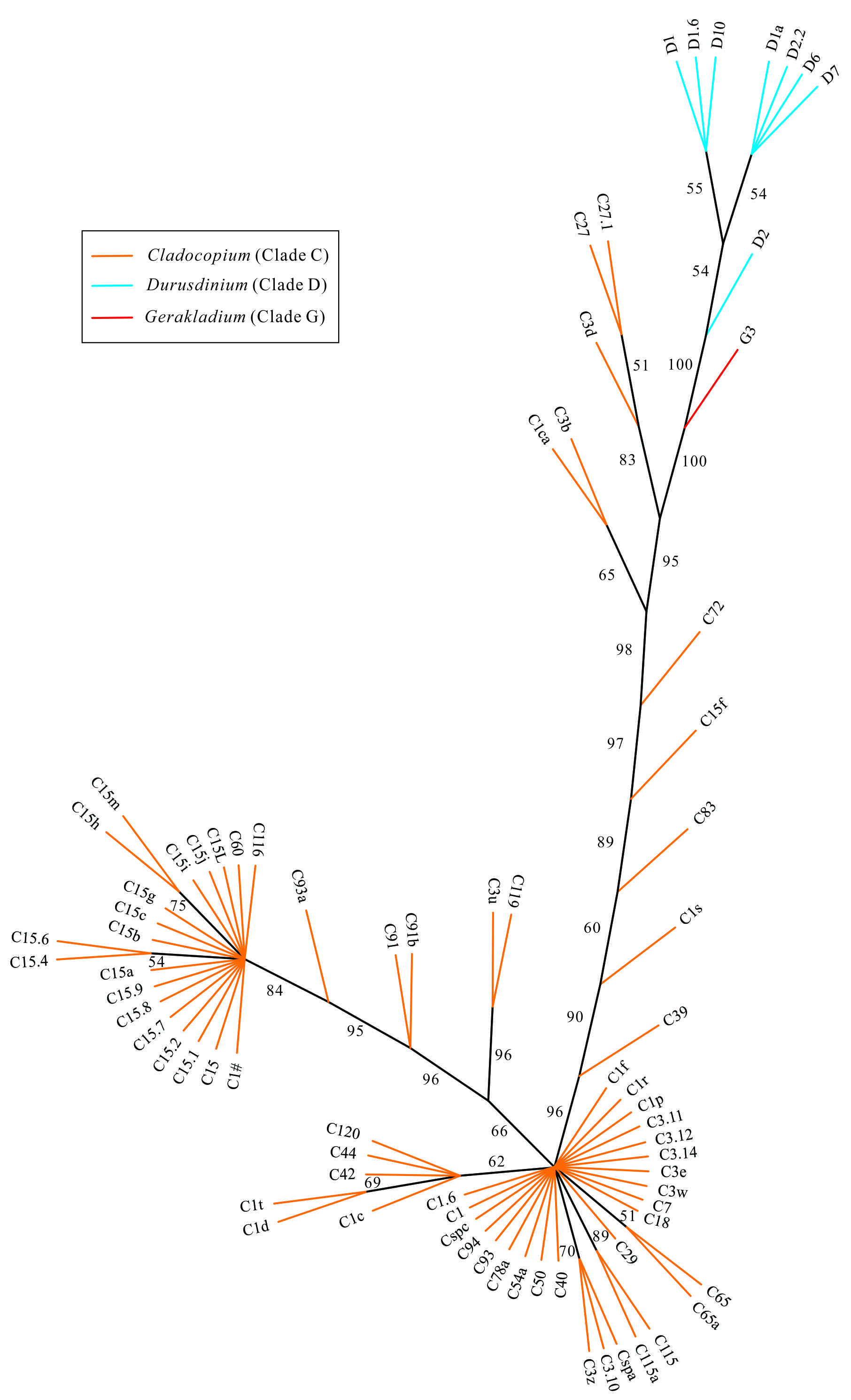

Supplement: Supplementary file 10 [file Image_7.JPEG]

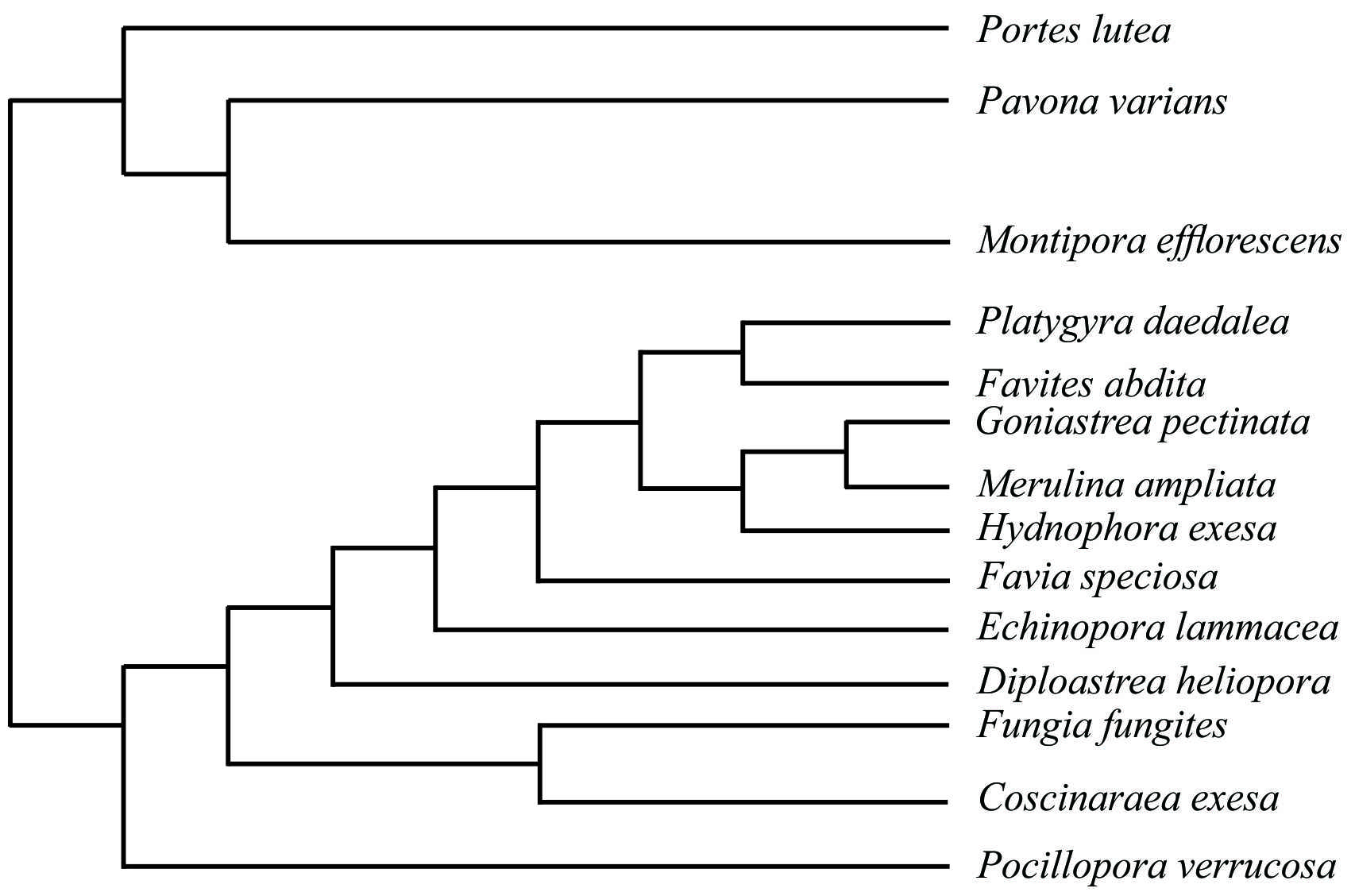

Supplement: Supplementary file 11 [file Image_8.JPEG]
